# End of life care during the COVID‐19 pandemic: A qualitative study on the perspectives of nurses and nurse assistants

**DOI:** 10.1002/nop2.1646

**Published:** 2023-03-08

**Authors:** Nertila Podgorica, Christine Rungg, Beatrice Bertini, Susanne Perkhofer, Christoph Zenzmaier

**Affiliations:** ^1^ Department of Nursing Health University of Applied Sciences Tyrol Innsbruck Austria; ^2^ Gruppo San Donato Hospitals Milan Italy; ^3^ Research and Innovation Unit Health University of Applied Sciences Tyrol Innsbruck Austria

## Abstract

**Aim:**

To explore nurses’ and nurse assistants’ experiences of providing end‐of‐life care during the COVID‐19 pandemic in Austria, Germany and Northern Italy.

**Design:**

A qualitative explorative interview study.

**Method:**

Data were collected between August and December 2020 and analysed using content analysis. Healthcare professionals (nurses (*n* = 30), nurse coordinators (*n* = 6) and nurse assistants (*n* = 5)) from hospitals (*n* = 32) and long‐term care facilities (*n* = 9) in Austria, Germany and Northern Italy were interviewed for this study.

**Results:**

Five main categories were identified as follows: (i) end‐of‐life care involves love and duty, (ii) last wishes and dignity of the patient, (iii) communication with the family, (iv) organizational and religious aspects and (v) personal emotions. Results indicate that more training and guidelines are needed to prepare nurses and nurse assistants for end‐of‐life care during pandemics.

**Public contribution:**

This research can help prepare nurses and nurse assistants for end‐of‐life care in pandemics and will be of value for improving the institutional and government health policies. Furthermore, it can be of value in preparing training for healthcare professionals patient–relatives.

## 1 INTRODUCTION

Coronavirus disease 2019 is a respiratory infectious disease caused by SARS‐CoV‐2 (World Health Organization, 2022). Health care professionals play a significant role in combating this health problem, particularly when patients suffer from severe symptoms that require hospital admission for treatment or even palliative care, as the main providers of end‐of‐life care. Several studies have demonstrated that nurses and nurse‐assistants experience pressure, burden, and emotional challenges concerning an increase in patient deaths (Hanna et al., 2021a, 2021b; Podgorica, Flatscher‐Thöni, et al., 2021; Podgorica, Pjetri, et al., 2021; Podgorica, Shabani, et al., 2022) and insufficient resources to provide care (Mehta et al., 2021; Podgorica, Shabani, et al., 2022; Podgorica, Zenzmaier, et al., 2022). Previous studies focused on emotional and psychological distress that affected health care professionals' well‐being (Huang et al., 2020, 2021; Mitchinson et al., 2021; Rathnayake et al., 2021). Health care professionals had to balance their duties and competencies with emergent choices and decisions in practice (Galehdar et al., 2020; Palacios‐Ceña et al., 2022; Turale et al., 2020). Patient‐centered care was rarely possible due to a lack of resources, and health care professionals often had to decide whom to provide care to (Jia et al., 2021; Palacios‐Ceña et al., 2022).

During the COVID‐19 pandemic, and in particular, in its initial phase, many hospitals and nursing homes applied strict admissions restrictions for visitors to limit disease transmission (Iness et al., 2022; Stratil et al., 2021). For instance, in Italy, in approximately half of the intensive care units (ICUs), visit patients were generally not allowed, and in the majority of the remaining ICUs, visits were only allowed for major events and mainly from the same family member (Langer et al., 2022). The visiting restrictions led to relatives' emotional issues that affected health workers. Nurses and nurse assistants could not give compassionate care to each patient, making them feel helpless to dying isolated patients without family and friends (Hanna et al., 2021a, 2021b; Palacios‐Ceña et al., 2022). Caring for patients and their relatives, nurses and nurse assistants tried to find alternatives to visits, such as video calls during the end of life (Hanna et al., 2021a).

The present study aimed to explore nurses' and nurse assistants' experiences while giving end‐of‐life care for patients with COVID‐19 in three EU countries (Austria, Germany, and Northern Italy). Nurses and nurse assistants were included in this study because they offered care as a team for patients with COVID‐19. Our findings are of value for improving institutional and government health policies and may help health workers prepare for future pandemics.

## 2 AIM AND OBJECTIVES

This study aims to explore nurses' and nurse assistants' experiences of providing end‐of‐life care during the pandemic. Nurses' and nurse assistants' relationships with patients and patients' relatives and their personal emotions while caring for patients with COVID‐19 in Austria, Germany and Italy were investigated.

## 3 METHODS

### 3.1 Design

A qualitative exploratory design using semi‐structured interviews and qualitative content analysis (Mayring, 2015; Polit & Beck, 2008) was most suitable for exploring individual experiences (Creswell & Poth, 2016; Polit & Beck, 2008). This study followed the Consolidated Criteria for Reporting Qualitative Research (Tong et al., 2007) and the standards for Reporting Qualitative Research (O'Brien et al., 2014).

### 3.2 Ethical considerations

This study followed the principles of the Helsinki Declaration (World Medical Association, 2008). All participants received written and oral information about the study and provided written and verbal consent. Informed consent of the study participants was obtained in written form. Once the data were collected, the participants were anonymized using identifiers (P01‐P41), and their names were removed from the transcribed data. The principal researcher kept the primary audio and video data in a secured file. The rest of the team worked with the pseudonymized transcripts. During the entire study, the participants were free to withdraw and request the deletion of their data at any time (e.g., via e‐mail) without any negative consequences. The Ethical Board of the principal researcher's institution approved this study (Nr: 2767; Date 10.06.2020).

### 3.3 Context and settings

This study was conducted in hospitals and nursing homes in three neighbouring European countries: Austria, Germany, and Northern Italy.

### 3.4 Participants' recruitment

Purposive sampling was used based on relevance to the research questions (Creswell & Plano Clark, 2018). The inclusion criteria included registered nurses and nurse assistants working in a hospital or nursing home and providing direct end‐of‐life care to patients diagnosed with COVID‐19. Participants were recruited via professional contacts. The researchers contacted them and sent the information about the study via e‐mail to seventy participants who expressed interest in participating.

### 3.5 Data collection

An interview guide to obtain information regarding nurses' and nurse assistants' experiences while giving end‐of‐life care for patients with COVID‐19 was developed based on the international literature (the interview guide is provided in Table S1). Two female nurse researchers (the first and the second author of this paper) conducted semi‐structured interviews in the participants' first language between August and December 2020. Interviews were conducted online via Zoom, video and audio and lasted between 50 and 120 min.

Interviews were transcribed verbatim using F4 software. Data were collected until the same topics started to be repeated, and nothing new came out; then, saturation was reached with 41 conducted interviews. The researchers took field notes to provide insights into participants' behaviour expressions and events during the interviews (Janghorban et al., 2014). Sociodemographic characteristics were collected by a questionnaire sent via e‐mail.

### 3.6 Data analysis

Transcripts were checked for accuracy by spot‐checking, repeatedly reading, and analysing four transcripts and field notes (MacLean et al., 2004). The original or translated transcripts were analysed using the Qualitative Content Analysis of Mayring as a methodical basis (Mayring, 2014, 2015) and with the help of MAXQDA2020 software (Kuckartz & Rädiker, 2019). Following the procedural model for inductive category formation, the research team worked through the material three times (Kuckartz, 2016; Kuckartz & Rädiker, 2019; Mayring, 2015). This process created inductive categories to structure the material with recurring topics (Table 1). The coded segments were first paraphrased, coded, and subsequently subsumed into (sub)categories (Table 1) to create a code system that fit the observations and the interview (Mayring, 2015). The authors performed the final coding separately, and minor disagreements were resolved within the team.

**TABLE 1:** Data generation process.

Segment	Paraphrase	Code	Subcategory	Category
To think that at the end of the day, these people do not even have the dignity to stay, to be clothed, for example, or the chance to be greeted by their own family, was hard. (P3)	Dying without family near and without usual rites means no dignity.	Dignity	Dying without dignity	Handling the last wishes and dignity of the patient
Uh, saying goodbye to patients who died was stressful and is an exaggeration. Nevertheless, it makes you think about how humanity has developed when you say goodbye via the iPad. Therefore, the relatives could not, it was the only possibility, and it is just slightly general, it is complicated there to say, it is a burden with the relatives. (P24)	Greeting the family members via iPad looks strange and not human, but it was better than nothing.	Last greetings	Saying Goodbye via Ipad	Communication with the family

## 4 RESULTS

Forty‐one interviews were conducted with nurses (*N* = 36) and nurse assistants (*N* = 5). The complete findings from the sociodemographic questionnaire are given in Table 2.

**TABLE 2:** Sample characteristics.

Participants [*N*]	41	
Country [*N* (%)]		
Austria	10	(24.4%)
Germany	8	(1.5%)
Italy	23	(56.1%)
Sex [*N* (%)]		
Female	31	(75.6%)
Male	10	(24.4%)
Age, years [mean (range)]	37.1	(24–60)
Educational level [*N* (%)]		
Nurse, BScN or MScN	36	(87.8%)
Nurse Coordinator	6	(14.6%)
Nurse‐assistant	5	(12.2%)
Working experience, years [mean (range)]	12.5	(1–36)
Work extent in % during pandemic [mean (range)]	89	(35–100)
Working place during COVID‐19 pandemic [*N* (%)]		
Hospital	32	(78.0%)
Nursing home	9	(22.0%)
Any differences in daily working [*N* (%)]		
Yes‐transfer to another station	3	(7.3%)
Yes‐different shifts	4	(9.8%)
Yes‐overtime	16	(39.0%)
No	18	(43.9%)
Number of beds in department [mean (range)]	25.1	(8–50)
Number of patients with COVID‐19 cared for[mean (range)]	34.7	(4–100)
Previous training on pandemics [*N* (%)]		
Yes	5	(12.2%)
No	36	(87.8%)
Working instructions before starting at COVID‐19 department [*N* (%)]		
Yes	23	(56.1%)
No	18	(43.9%)
Protective equipment [*N* (%)]		
Sufficient	32	(78.0%)
Insufficient	9	(22.0%)
Number of people living with [mean (range)]	1.9	(0–4)
Spiritual/religious person [*N* (%)]		
Yes	23	(56.1%)
No	13	(31.7%)

Five main categories were identified and summarized through the qualitative content analysis in Table 3.

**TABLE 3:** Categories and subcategories.

Category	Subcategory
End‐of‐life care is love and duty	Being human and never letting the patient die alone
	Adapting care
	Diminished care
Last wishes and dignity of the patient	Dying without dignity
	Caring for isolated patients
	Supporting patients' and relatives' wishes
Communication with the family	Virtual communication Communicating the death of a patient to relatives
Organizational and religious aspects	Organizational problems
	Triage
	Religious last rites
Personal emotions	Coping with exceptional situations
	Emotional burnout

### 4.1 End‐of‐life care is love and duty

All participating nurses and nurse assistants described end‐of‐life care for patients with COVID‐19, including being near to them and not letting patients die alone. Given strict visiting limitations or bans, it was essential for some participants to act like family members in the very last moments of a patient's life.

#### 4.1.1 Being human and never letting the patient die alone

Participants took on the role of family members while the patients were at the end of their lives and tried to find time to be there: “*What we tried to do is never let anyone die alone, okay? Therefore, the health workers did what the relatives would do at the time of death. When the patient's death was confirmed, it was also communicated to the relatives to know that these people did not die alone*.” (P3).

“Being human” was described as being there for the patient, respecting them while alive and after death. One of the Italian participants stated that the worst experiences were the ways in which many patients died, with great difficulties, pain, and alone. “*When we knew that there was nothing more to do, we tried to meet the needs of the patient, the preferences more than anything else, so we tried to satisfy them in the best way*.” (P12).

#### 4.1.2 Adapting care

Despite being in a chaotic situation facing a pandemic without feeling prepared, participants tried to adapt care as much as possible to satisfy patients' needs. One participant emphasized that it was a moral obligation to care for patients with love and assist them until the last moment of life: “*People died there as if they were flowers, those withered flowers that died in two seconds, so every ward was chaotic. It was hard, but with so much love from all my colleagues, passion, and a sense of duty. I have a nice big task, and you only gave them your hand to accompany them to the mortuary, and that is it*.” (P18).

Many interviewees expressed compassion for patients or reported simple acts, such as playing patients' favourite music in the background, dabbing them with their favourite perfume, or simply holding their hands while dying. This was mainly reported in nursing homes, as participants had built relationships with patients over time.

Due to stringent visitor admission restrictions applied during the first wave of the pandemic, even close family members were frequently not allowed to visit patients during the pandemic. Participants used different strategies to handle this situation, such as enabling virtual communication or trying to provide as much personal support and companionship as possible. “*Another sad thing was the separation of the relatives. I had to be a nurse, a wife, a mother, a lover, and everything for the patient. That was the most heartbreaking thing, the most heartbreaking thing I could ever feel. I hope I will never feel that again*” (P8, referring to the death of a colleague who died one month before his retirement).

#### 4.1.3 Diminished care

Many participants reported that it was frequently impossible to provide needed care due to lack of time: “*It was a very, very lonely death, I think. You probably know that dying in the hospital is usually very lonely anyway. However, there are still the exceptions when you somehow have time to (.)—I do not know—again to provide a little relief, exactly to provide comfort, a little assistance, and that was now hardly possible because of the workload*” (P34).

Interviewees felt guilty, useless, and helpless when they found themselves unable to provide appropriate care due to a lack of medical and technical equipment, competence, and knowledge about COVID‐19. They reported episodes where medication was interrupted, and patients were left to die, which they considered unethical: “*They did not get anything anymore, they did not get any painkillers or anything, and that is irresponsible and unethical. (…)*” (P28).

### 4.2 Last wishes and the dignity of the patient

#### 4.2.1 Dying without dignity

Almost all participants reported how patients died without dignity. It was frequently impossible to perform the last rituals and to dress the dead: “*To think that at the end of the day these people don't even have the dignity to stay, to be clothed, for example, or the chance to be greeted by their own family, was hard*” (P3, referring to deceased patients whose corpses were immediately transferred to the morgue and cremated).

Participants pointed out how the dead bodies were treated, packed in bags, and labelled. Some of them expressed difficulties having patients die without relatives: “*Then (…) we did not do anything with dignity. We did not let them see the face of their relatives, the children, or nobody. Even the funeral and everything was done without anyone, only by the agencies. Therefore, this has nothing to do with a dignified death, zero; I am sorry*” (P13).

Whereas all Italian participants reported the absence of family members, restrictions were less drastic in Germany and Austria, where close relatives were often allowed to be present at the very last moments or after the death of the patients.

#### 4.2.2 Caring for isolated patients

The isolation regime caused difficulties in the process of care. No one was allowed to stay in the rooms with patients with COVID‐19 and health care personnel were only for basic care, making the participants feel like they were abandoning the patients who often died isolated. Some participants found that giving care without touching or talking was extremely hard: “*We had a dying resident who needed the closeness, the touch. He had tears in his eyes and would have just needed someone to give him a talk, one hand and not through a glove, through the protective suit, through the whole thing*” (P31).

#### 4.2.3 Supporting patients' and relatives' wishes

Participants felt terrible when they could not respect patients' or their family's wishes. In one reported case, even the living will of the patient was not respected: “*She was very (.) optimistic and had told me ‘Please, allow no one to intubate me; (..) it was written; she did not allow it. The notary stated it. Therefore, they knew it, but they intubated her. I felt so sorry when she died. I was irritated when I heard she was intubated; I was upset*” (P39).

Some patients even died from suicide. A participant reported a patient note stating, “*‘Sorry, I wanted to die.’ … removed his oxygen helmet and was later found deceased*” (P11).

Due to visitor restrictions, the situation in nursing homes was very burdensome for residents not being allowed to see their relatives for weeks and months. Some residents were desperate, exemplified by the suicide of a 94‐year‐old resident in an Austrian nursing home: “*And again, a lockdown, and it was already everything normal, and then he died from suicide. He had strangled himself on the bed rails; what a person can do! Yes (.), and he had not seen his family, his children, for the whole time before that*” (P26).

#### 4.2.4 Communication with the family

Most participants highlighted the importance of family‐patient communication despite visitor restrictions.

#### 4.2.5 Virtual communication

Isolation required new ways of interacting with relatives. Video calls were crucial, as patients had not seen their relatives for days or weeks. Sometimes relatives did not want to see their loved ones in bad condition, or patients did not want to make video calls. “*Because of the bad clinical conditions in which the patients were, they often didn't want to be seen by their relatives, even via video calls*” (P12).

Virtual communication was perceived as necessary during the last moments of patients' lives, sometimes assisting family‐patient communication. However, this kind of communication was also considered strange. “*Uh… when saying goodbye… patients, who died and it was (..) stressful is perhaps an exaggeration, but it makes you think about how humanity has developed when you say goodbye via iPad*” (P24).

Despite all efforts, virtual communication was not always possible, so many patients died without saying goodbye to their loved ones. Patients died very quickly during the first pandemic wave, particularly in Italy, without knowing they were dying or having time to greet their loved ones.

#### 4.2.6 Communicating the death of a patient to relatives

Communicating patients' death to relatives by phone or video was perceived as something beyond the difficulties, leading to tragic and emotional situations: “*Unfortunately, there was no presence of family members during the death, and therefore at the time when the patient was declared dead, the family members were not there; they were notified by phone. The body was taken to another department, to the morgue department, and I know that the family members could not approach; they could only see the body on the day of burial*” (P21).

### 4.3 Organizational and religious aspects

#### 4.3.1 Organizational problems

Most participants had to face organizational problems. Governments, regions, or institutions organized end‐of‐life care, medical techniques, equipment, training, etc., Respondents from Italy indicated that they had instructions for treating the dead bodies: “*The protocols changed, I don't know, even just for the dead, that is one… the dead were disinfected with bleach, put in a bag, sent to the morgue, and then; they were all cremated*” (P3).

Participants mentioned issues related to the lack of training and organization: “*There is something wrong in the system, and now it is just becoming apparent through the corona virus. Yes, everything that we missed in the last decades, including better training of personnel, training, um, is now somehow taking revenge*” (P38).

#### 4.3.2 Triage

In Italy, participants needed to admit patients to ICUs and intubate patients due to deteriorating conditions to continue intensive care. Limited resources resulted in restrictive selection criteria: “*The worst experience was of a patient who practically died fully conscious. There was no place for him in the ICU because he was too old, and we did not intubate him but let him go with the CPAP helmet still on his head. That was not good, and I remember that*” (P11).

Participants were involved in decision‐making to implement restrictive measures related to care, significantly affecting patients. They felt useless for being unable to tell the truth and offer appropriate qualitative care to patients. “*While the anesthetist and a colleague of mine were intubating one patient, the patient next to him said, “I have three children at home. Why did you only intubate him and not me as well?” that was the most difficult part because these are situations where you cannot answer*” (P11).

Participants from Austria and Germany did not report similar experiences, as triage was unnecessary in their institutions.

#### 4.3.3 Religious last rites

Italians placed great significance on religion; they saw many people dying and had stringent rules due to the pandemic. Participants gave care and played the role of a family member or even a priest. Participants from Austria and Germany mentioned the presence of a priest. In Italy, regional regulations prevented most deceased from receiving religious last rites, as none could enter the ICU, other COVID‐19 departments, or nursing homes. “*Apart from that, everyone could pray within themselves, and there were no priests. It was impossible; you could not get a priest in to bless them, and (…) the funerals were done as quickly as possible. Therefore, all that spiritual and human part was missing slightly because of this emergency*” (P22).

### 4.4 Personal emotions

During the interview process, all participants reported having a deep emotional state.

#### 4.4.1 Coping with exceptional situations

Many participants reported emotional moments while caring for COVID‐19‐infected patients and witnessing patients' death. In particular, the handling of the deceased was very memorable, as reported by an Italian nurse: “*…all their personal things were thrown away, they could not be given to the relatives, or they were burnt… After we had prepared the patient, they were put in this airtight bag, and they were put on the stretcher, and we put a sheet with the name written on it. Writing the names on the sheet was truly… you felt exhausted because you said, ‘It can't be possible’*” (P4).

Another participant expressed her experiences of facing moments of preparation or disinfection of the corpse alone. “*Eh, that was (…) one of those moments difficult to forget because it was a procedure that touched me psychologically. We had to disinfect the corpse, and we would then put the body in a clean sheet, and then we knew that nobody would ever see it again*” (P18).

#### 4.4.2 Emotional burnout

Many participants cried during the interview and complained of being overwhelmed by the situation. A nurse referred to patients dying without their relatives being around. The team tried to enable video calls and to provide as much support as possible: “*As far as I am concerned, what my colleagues and I did was the maximum, and when someone died, there was that moment of silence. I do not know, it was silence, even if the patient was not one of your relatives, you felt (…) like losing somebody you know. There was that moment of silence that you remained quiet (…) I do not know, ‘321 died,’ that is, you did not even know who the patient was, or, you could know them because if you had done the previous shift, you knew which patient was not okay. However, as you arrived, you already saw the air (…) that was there, which made you understand that something had happened, therefore, I think that each of us put our heart toward the patients, we put our heart into this situation*” (P23).

A German nurse working at a nursing home referred to the emotional impact of experiencing residents' death: “*Um, I think, you know, especially for me as a young colleague, I think it affects me even more. It is not that I wake up at night and think of these humans. However, I talked about it with my friends who work in the health care system, and we helped each other with pseudo‐psychological care. Then, it is okay*” (P34).

Another participant described, “*I don't have a problem with dying and taking care of them, rewashing them, but putting them in the bag feels wrong, like I'm a murderer or something; that's such a weird feeling*” (P36).

Interviewing some participants was difficult due to their emotional state; as one of the interviewees said, “*Please don't end the interview now, because I want you to listen to me until the end*.” While the German and Austrian participants tried to report calmly and objectively, the participants from Italy were sometimes very stirred up and emotional due to the re‐experiencing of critical situations in the interviews. Sometimes the interview ended, and the recording stopped, but the interviewees kept talking for more hours.

## 5 DISCUSSION

This study contributes to the growing research on the COVID‐19 pandemic (Al‐Amer et al., 2022; Arcadi et al., 2021; Catania et al., 2021; Simeone et al., 2022). The topics described in this study, end‐of‐life care involving love and duty, patient's last wishes and dignity, communication with family, organizational and religious aspects, and personal emotions, show that nurses and nurse assistants who cared for patients with COVID‐19 encountered many challenges in providing quality care to patients at the end of life.

In this and other studies, the high mortality rate of patients with COVID‐19 triggered many participants' deep sense of grief. They described losing one patient after another (Gordon et al., 2021; Montgomery et al., 2021; Podgorica, Zenzmaier, et al., 2022). Participants dealt with patients who talked to them for a minute, then decompensated and died shortly thereafter, despite all efforts to save their lives (Podgorica, Zenzmaier, et al., 2022; Robinson & Stinson, 2021; Simeone et al., 2022).

Although grief was exacerbated by the absence of these patients' relatives at the bedside due to facility regulations, participants expressed compassion. They acted as if they were the relatives of the patients accompanying them to the very end. The research from Arcadi et al. (2021), and Carnesten et al. (2022) emphasizes the importance of compassion concerning well‐being and care based on love, empathy, and the intention to alleviate suffering and support the health of patients (Arcadi et al., 2021; Carnesten et al., 2022).

During these critical moments, despite all the difficulties in providing end‐of‐life care, the study participants tried to be humane and respect the patients by staying close to them, not letting them die alone, and fulfilling their duties and responsibilities. In this context, Liu et al. (2020) and Mitchinson et al. (2021) also reported that health care workers performed their tasks by not letting patients die alone and offering them a death as bearable as possible (Liu et al., 2020; Mitchinson et al., 2021).

Considering their professional and moral obligation to care for the patients, the nurses and nurse assistants in this study, despite their lack of evidence‐based knowledge about the disease, took great responsibility by adapting the care and supporting the patients until their last moments. The patterns found in this study are similar to the findings identified in various studies worldwide (Copel et al., 2022; Smeltzer et al., 2022). Indeed, (Specht et al., 2021), who found that nurses have a strong sense of responsibility and professional duties, can support our findings. Likewise, Scrymgeour et al. (2020) studied participants demonstrated the ability to take responsibility, adopt care, and cope despite various pandemic obstacles (Scrymgeour et al., 2020).

Despite participants' best efforts, the pandemic affected their ability to provide high‐quality care. Being unable to provide the desired quality of care, not knowing if treatment protocols were appropriate, wondering if they had done enough to save patients' lives, lacking the necessary time, and keeping families away from the bedside of the dying led to intense feelings of guilt and helplessness, as well as ethical conflicts.

Sperling (2021) also reported in their study that ethical conflicts arose when nurses could do nothing to save their patients' lives and could not provide optimal care for COVID‐19 patients due to limited treatment options and resources (Sperling, 2021). Other studies described the inability to offer COVID‐19 patients the needed care (Smeltzer et al., 2022) and feelings of fear, guilt, and helplessness in the face of an unknown disease (Arcadi et al., 2021; Podgorica, Zenzmaier, et al., 2022; Simeone et al., 2022).

Given the strict rules on the use of protective equipment and the limited opportunities to touch and talk to isolated patients, the nurses and nurse assistants in this study struggled to provide good care. This finding is consistent with other studies in which nurses found it challenging to touch, care for, and build relationships with isolated patients (Hanna et al., 2021a, 2021b; Onwuteaka‐Philipsen et al., 2021; Rathnayake et al., 2021).

Hence, new protocols were established to treat the corpses to prevent the spread of COVID‐19. Participants prescribed an undignified death for patients without the presence of family members, in isolation, and with limited burial ceremonies. Selman et al. (2021) supported these findings by reporting that people died in isolation, without burial or rituals, yet experienced the ultimate injustice of dying without their loved ones to mourn for them (Selman et al., 2021).

Similar to Mannix et al. (2020), participants in the present study had tremendous difficulties fulfilling the wishes of patients and their families (Mannix et al., 2020). Because of the complex circumstances mentioned above, this finding highlighted participants' limitations and obstacles in communicating with isolated (and dying) COVID‐19 patients and their families. Similar conclusions were reached by other reseachers, all of whom stated that social distancing and restrictive measures, as well as fear of infection, affected health care professionals' lives, as they could not communicate enough with dying patients and their relatives (Copel et al., 2022; Dempsey et al., 2022; Langer et al., 2022; Thrysoee et al., 2022). Throughout these situations, nurses relied upon virtual communication technology to enable families to connect and communicate with patients while their loved ones were dying. Copel et al. (2022), Dempsey et al. (2022), and Langer et al. (2022) asserted that online communication could sustain health care professionals during a pandemic (Copel et al., 2022; Dempsey et al., 2022; Langer et al., 2022).

In line with previous research, our findings emphasize the need and importance for training on communication with isolated patients and family members during end‐of‐life care in the event of a pandemic (Dempsey et al., 2022; Podgorica, Pjetri, et al., 2021; Thrysoee et al., 2022). Therefore, nurses and assistant nurses need support and facilities for virtual technologies and training.

During the COVID‐19 pandemic, the number of patients who required intensive care treatment outnumbered the number of intensive care beds in many regions, even in industrialized nations. Consequently, the restrictions and guidelines imposed by governments and authorities worldwide have generated many organizational problems, and triage has sometimes become necessary (Akalpler & Okumus, 2018; Arcadi et al., 2021; Catania et al., 2021; Muz & Erdoğan, 2021). Challenges related to the organization of end‐of‐life care for patients with COVID‐19 within the institution, time management, or training were reported to have forced some of our study participants to decide whom to care for and how much time to spend with each patient. These participants were very emotional while describing the triage process during the interviews.

Previous studies reported similar findings when health care professionals felt unable to care for all patients equally due to resource issues and had to choose between the well‐being of one patient over another. In line with other studies, the triage criteria for prioritizing patient care based on age or health state during the pandemic caused ethical conflicts and dilemmas for participants (Booke & Booke, 2021; Palacios‐Ceña et al., 2022; Smithard & Haslam, 2021).

Many people consider religious personnel, such as priests, the professionals most skilled in spiritual care and important in dealing with the spiritual beliefs of seriously ill patients, especially at the very end of their lives.

In the same direction, in normal conditions, the family provides the majority of spiritual care to the patients who are living their last moments at home or the hospital, as previous studies have shown (Rathnayake et al., 2021). In this context, health professionals are responsible for establishing this link between the family, religious personnel, and the patient, creating a safer and more comfortable environment (Nascimento et al., 2016).

However, in this study, the restrictions on visits by family members and religious personnel during the pandemic increased the responsibility of the study participants, particularly in Italy, who reported acting like relatives of the patients and sometimes fulfilling the role of religious personnel by performing last rites and praying for the dying patients. In this context, Rocío de Diego‐cordero et al. (2022) found that some of the participants gave (spiritual) religious last rites to the patients, and they suggested including religious rituals (spiritual care) as a coping strategy for future pandemics (de Diego‐cordero et al., 2022).

Health care professionals who face trauma, death, and disruption are at risk of developing stress and traumatic disorders (Specht et al., 2021). The constant variability of clinical situations during COVID‐19, the high mortality rate, and the poor outcomes for surviving patients contributed to the stress level of health professionals. The results of this study, similar to other evidence, showed that the care of dying patients was experienced emotionally by the participants, who reported anxiety, agitation, stress, and sleep disturbances (Copel et al., 2022; Galehdar et al., 2020; Podgorica, Zenzmaier, et al., 2022; Smeltzer et al., 2022). Preparation and disinfection of corpses resulted in emotional overload or even burnout among respondents, some of whom felt guilty and unable to help patients. Similar findings were reported by Copel et al. (2022), Mulyadi et al. (2022), and Smeltzer et al. (2022), who pointed to the personal distress nurses felt as their patients' conditions deteriorated or were withdrawn from life support. In the midst of these difficulties, participants found ways to cope with emotional distress. They reported that conversations with friends, colleagues, and family served as therapy for them. Some of them mentioned that they cried a lot, talked, and were comforted, found support from their colleagues, helped each other, and developed solidarity. Accordingly, Thrysoee et al. (2022) mentioned solidarity and mutual support between nurses caring for patients with COVID‐19.

Other studies have also shown the importance of the family and team supporting each other and caring for each other's mental health (Al‐Amer et al., 2022; Podgorica, Zenzmaier, et al., 2022). There is a need to provide psychological support and care for those affected.

### 5.1 Strengths and limitations

Our results may affect the improvement of care and management during pandemics. This is the first study that provides information on nurses' and nurse assistants' experience in end‐of‐life care during the COVID‐19 pandemic in different settings (hospitals and nursing homes) in three different EU countries. It contributes to the literature on caregiving during pandemics. Most of our sample were nurses because they were health care professionals with more extended contact with end‐of‐life patients. The transferability of the findings is a weakness of this study. Future studies should consider the experience of all health care professionals involved in providing end‐of‐life care during pandemic situations.

Our findings are limited to nurses and nurse assistants working in Austria, Germany, and Northern Italy during the COVID‐19 pandemic. Future research should investigate health care professionals' experience in different countries, including non‐EU countries.

## 6 CONCLUSION

Nurses and nurse assistants have faced significant professional and emotional difficulties in providing end‐of‐life care during the COVID‐19 pandemic. This is related to the increased number of end‐of‐life patients, reduced and untrained staff, inappropriate institutional management of the pandemic, and the isolation of the patients from their relatives.

Training and guidelines are needed to prepare nurses and nurse assistants for end‐of‐life care during pandemics. These should also include preparation on how the health care professionals can deal with patients who do not have relatives who visit, keeping up morale, and a component of coping during a pandemic since it could happen again. Hospitals and nursing homes should review policies to prevent emotional burnout and offer incentives to retain trained staff during emergencies.

## AUTHOR CONTRIBUTIONS

Study conception, NP; design, NP, CZ, CR; data extraction, NP, CR, BB; analysis, NP, CR, CZ; quality appraisal, NP, SP; NP, led the drafting of the manuscript, with the contribution of CR and CZ. All authors revised the manuscript and approved the final version before submission.

## FUNDING INFORMATION

The author(s) received no financial support for this article's research, authorship, and publication.

## CONFLICT OF INTEREST STATEMENT

The author(s) declared no potential conflicts of interest concerning this article's research, authorship, and publication.

## SUPPLEMENTAL MATERIAL

Supplemental material for this article is available online.

## Supporting information


Table S1

## Data Availability

Data are not publicly deposited but are available (University of Applied Sciences Tyrol, Austria) upon request to the authors.
