# Supplementary material for: End of life care during the COVID‐19 pandemic: A qualitative study on the perspectives of nurses and nurse assistants
Source: Nurs Open. 2023 Mar 8;10(6):3881–91. doi: 10.1002/nop2.1646 (PMC10170946; doi:10.1002/nop2.1646)
Supplement: Supplementary file 1 — Table S1 [file NOP2-10-3881-s001.docx]

**Table S1:** Interview Guide

| Questions | |
| --- | --- |
| 1. To start the interview, can you tell me about your daily work as a nurse during the crisis? How did your daily work look like? | |
| - What was your working situation? - What was different about it compared to your normal working situation? - Did you feel motivated to work at this time or was it difficult for you to fulfil your job duties? Why? | - Can you please tell me more about that? - What do you mean by that concretely? |
| 1. How did you feel when caring for Covid-19 patients? | |
| - Do you find that the care of Covid-19 patients is a special situation? - What were the major challenges in/during the care of patients with Covid-19? (personal problems, problems with the organization of the working process, institutional strains, lack of materials) - If your family members were no longer touching or interacting with you, how did you feel? - If family members discouraged you to work/tried to stop you from going to work, how did you respond? How did you feel and react to them and your job? - Did you and your colleagues keep each other at distance? (e.g., no hugging or kissing) How did that feel? How did it affect you professionally? | - Why do you think about that? - What is special about it? - What are the differences? - How did you handle this situation? - Did this help/did you feel better afterward? - Do you think there are better ways that you could have handled the situation? |
| 1. What difficulties and problems did you face during the crisis? | |
| - What kind of problems occurred? - What was your worst or your most painful experience during the crises? - Were there any negative impacts on you as a person or your personal life? - Have you ever been afraid during the crisis? Of what? Did you feel lonely or stressed? Why? - Did you have any arguments with patients or their relatives? (Why?) - Did you have any arguments with other people, e.g., colleagues, your family or friends (why?) - Was anyone from your work environment or any colleague of yours infected with the virus? - If a nurse had been stigmatized, how did it make you feel? How did you react? - If a nurse who was caring for a patient or was working with a colleague who died of (because of) COVID-19; what effect did it have on you? How did you manage to continue working (or not) in such a situation? | - Anything else? - Any other problems? - How did you feel in this situation? - How did you handle this situation? - Did this help/did you feel better afterward? - Do you think there are better ways that you could have handled the situation? |
| 1. In the media, nurses were portrayed as heroes and received much applause for their work. What do you think about this? How do you feel about it? | |
| - Are you proud of being a nurse? - Are there people in your surroundings who are proud of you being a nurse? - Do you think this crisis has led to strengthening the image of nurses? - Do you like the image of nurses as heroes? Do you find it appropriate? | - Does this have any influence on your current situation? - What do you mean by that concretely? |
| 1. For you personally, what were the (some) positive aspects about the crisis? | |
| - Is there anything good about the crisis? - What did you learn from your experiences? - Do you also have fond memories from that time? Which ones? | - Can you please tell me more about that? - What do you mean by that concretely? |
| 1. Based on your knowledge and experience, what would you do or change, if a comparable situation arose? | |
| - What advice would you give to a young nurse, working for the first time in such a pandemic crisis? - Would you work as a nurse again if there were another crisis? - What would you change to improve the situation? - What should other people/politics or institutions change? | - What would you do? - Are there things you will not do again? What kind of things? Why? |
| 1. Is there anything else you would like to share, anything that is important to you and has not been mentioned in the interview thus far? | |
